# Traits associated with central pain augmentation in the Knee Pain In the Community (KPIC) cohort

**DOI:** 10.1097/j.pain.0000000000001183

**Published:** 2018-02-09

**Authors:** Kehinde Akin-Akinyosoye, Nadia Frowd, Laura Marshall, Joanne Stocks, Gwen S. Fernandes, Ana Valdes, Daniel F. McWilliams, Weiya Zhang, Michael Doherty, Eamonn Ferguson, David A. Walsh

**Affiliations:** a Arthritis Research UK Pain Centre, Nottingham, United Kingdom; b Division of Rheumatology, Orthopaedics, and Dermatology, School of Medicine, University of Nottingham, Nottingham, United Kingdom; c Arthritis Research UK Centre for Sports, Exercise, and Osteoarthritis, Nottingham, United Kingdom; d NIHR Nottingham Biomedical Research Centre, Nottingham University Hospitals, NHS Trust, Nottingham, United Kingdom; e School of Psychology, University of Nottingham, Nottingham, United Kingdom

**Keywords:** Knee pain, Phenotypes, Central mechanisms, Quantitative sensory testing

## Abstract

This study aimed to identify self-report correlates of central pain augmentation in individuals with knee pain. A subset of participants (n = 420) in the Knee Pain and related health In the Community (KPIC) baseline survey undertook pressure pain detection threshold (PPT) assessments. Items measuring specific traits related to central pain mechanisms were selected from the survey based on expert consensus, face validity, item association with underlying constructs measured by originating host questionnaires, adequate targeting, and PPT correlations. Pain distribution was reported on a body manikin. A “central pain mechanisms” factor was sought by factor analysis. Associations of items, the derived factor, and originating questionnaires with PPTs were compared. Eight self-report items measuring traits of anxiety, depression, catastrophizing, neuropathic-like pain, fatigue, sleep disturbance, pain distribution, and cognitive impact were identified as likely indices of central pain mechanisms. Pressure pain detection thresholds were associated with items representing each trait and with their originating scales. Pain distribution classified as “pain below the waist additional to knee pain” was more strongly associated with low PPT than were alternative classifications of pain distribution. A single factor, interpreted as “central pain mechanisms,” was identified across the 8 selected items and explained variation in PPT (*R*^2^ = 0.17) better than did any originating scale (*R*^2^ = 0.10-0.13). In conclusion, including representative items within a composite self-report tool might help identify people with centrally augmented knee pain.

## Abstract 2

Supplemental Digital Content is Available in the Text.

Self-report items measuring 8 pain-related traits represent a unifying construct. This construct, and items, are significant correlates of quantitative sensory testing indices for centrally augmented knee pain.

## 1. Introduction

Knee pain is a major source of disability, and in people aged over 50 years is most commonly attributed to osteoarthritis (OA).^60^ Osteoarthritis pain is perceived as originating from the joint, often associated with structural changes or inflammation, and exacerbated by joint loading and movement. However, OA pain is often troublesome even in the absence of severe radiographic change,^24^ and might persist after removal of the peripheral nociceptive drive, with persistent pain being reported by 10% to 20% of people after total knee replacement for knee OA.^4,79,80^ Evidence from mechanistic (ie, experimental pain testing and functional neuroimaging studies)^25,30,33,59,60,69^ and therapeutic trials^11,29^ indicates that the central nervous system (CNS) might amplify neural signalling and influence OA knee pain sensitivity, leading to central pain augmentation.^42,78^ Optimal management of OA knee pain therefore requires that underlying pain mechanisms be identified in each individual.^3^

Quantitative sensory testing can indicate changes in pain sensitivity. Pressure pain detection thresholds (PPTs) might be reduced at a site of clinical pain, suggesting neuronal sensitization of the affected area. More widespread increased sensitivity at pain-free control sites is suggestive of altered pain processing in the CNS.^16,31^ In animal models of OA, pain sensitivity (reduced withdrawal thresholds to punctate stimulation) at a site distal to the affected knee (hindpaw) is characterized by spinal hyperexcitability of neurons innervating sites distal to the affected joint.^23,56,63,64^ Furthermore, pain sensitivity distal to the affected joint in people with OA has been associated with changes to descending pain control mechanisms,^33^ as has more widespread pain (WSP) in people with fibromyalgia.^5^

Individual differences in distinct observable traits (phenotypes), measured by questionnaires addressing depression, anxiety, catastrophizing, neuropathic-like pain, or WSP, have been associated with knee pain severity.^10,16,35,39,62,67,68^ Each of these traits might also be associated with markers of central pain mechanisms.^6,7,36,45,46,49,51,62,71^ High scores on these questionnaires, and low PPTs, have each predicted poor outcome after treatment directed to the painful joint,^2,59,60,79,80^ raising the possibility that treatments directed to central pain mechanisms might be useful for those patients. Using a full battery of existing questionnaires plus PPT measurement would be resource-intensive during normal clinical encounters. A concise composite self-report tool is needed to help identify people with centrally augmented knee pain.

We hypothesise that each of these traits might reflect aspects of central pain mechanisms. By combining evidence from expert opinion and statistical analysis of questionnaire data from a community-based study in people with knee pain, we aimed to identify a concise, yet psychometrically reliable and valid set of self-report questions that measure a phenotypic trait associated with central pain augmentation, as indicated by reduced PPT at the proximal tibia, a site distal to the painful knee.

## 2. Methods

### 2.1. Study population

Participants aged 40 years or older provided baseline data within the Nottinghamshire community-based Knee Pain and Related Health in the Community study (KPIC) cohort study.^22^ Questionnaires factor structure was confirmed using data from 2512 participants who reported current knee pain (61 ± 10 years, 57% female). A purposive subset of KPIC participants (n = 420) underwent further clinical, PPT, and radiographic assessments.^22^ This subset comprised people with no knee pain (n = 98), or pain for <3 years (n = 219) or >3 years (n = 103). The KPIC study protocol (clinicaltrials.gov portal: NCT02098070) was approved by the Nottingham Research Ethics Committee 1 (NREC Ref: 14/EM/0015) and all participants provided informed written consent.

### 2.2. Self-report questionnaires

Presence of current knee pain was determined by response to the question: “Have you had knee pain for most days of the past 1 month?”^61,74^

Participants reporting knee pain indicated the affected knee if unilateral, or the worst affected knee if bilateral.

The KPIC baseline survey included established self-report questionnaires for neuropathic-like pain (painDETECT modified for use in people with knee OA),^39^ intermittent and constant OA knee pain (ICOAP),^37^ catastrophic thinking (Pain Catastrophizing Scale [PCS]),^72^ and anxiety and depression (Hospital Anxiety and Depression Scale [HADS]).^81^ Traits of fatigue, cognitive impact,^65^ and pain distribution^40^ were each measured by single items. Rasch‐transformed questionnaire scores were used when previously validated in knee pain cases (painDETECT and ICOAP),^37,39^ otherwise nontransformed scores were used (HADS and PCS). Items were coded so that higher scores represented greater pain or distress.

Pain distribution was captured using areas shaded by the participant on a body manikin. The manikin was coded according to shading in 7 and 25 topographical areas.^15,77^ Pain distribution was also categorized using American College of Rheumatology Widespread Pain (ACR's WSP) criteria,^77^ and based on the presence or absence of pain (1) contralateral to the index knee, (2) above the waist, (3) below the waist, or (4) axial.

### 2.3. Pressure pain detection thresholds

The PPT was measured using a hand-held pressure algometer with a circular (1 cm^2^) padded-tipped probe connected to a computer (HP ProBook 4520s), with outputs computer analysed by dedicated software (Somedic AB, Sweden). Pressure was applied with a standardised 30 kPa/s ramp until the participant indicated by pressing a button, a change from pressure to pain sensation. Participants were familiarised before testing by twice PPT testing on a fingernail of the dominant hand. Each PPT testing cycle was conducted at the sternum (3-cm caudal to the sternal notch), the medial and lateral tibiofemoral joint lines adjacent to the patellar ligament of each knee, and the proximal tibia (5-cm distal to the tibial tuberosity of each leg). The PPT cycle was repeated 3 times with a 2-minute rest period between each cycle. Pressure pain detection threshold values (kPA) for each site were averaged across the 3 cycles. Pressure pain detection threshold assessments for each participant were undertaken using a standardized protocol by 1 of 2 trained researchers, blinded to participant characteristics including pain status.^22^

Raw PPT values were not normally distributed, thus PPTs were logarithmically transformed before statistical analysis to achieve normality of the data, and normality confirmed using the Shapiro–Wilk test.

Pressure pain detection threshold values served as a reference test during receiver-operating curve analysis to identify the number of painful sites other than the knee, reported on the body pain manikin that is indicative of central pain mechanisms. Preliminary analysis demonstrated no significant differences in PPT between participants with or without knee pain, and therefore, standardized *z*-scores were computed from log PPT data for all 420 participants. Pressure pain detection threshold values below the 10th percentile (*z* > 1.28) were classified as abnormally increased sensitivity (gain-of-function) at the measured site.^14^ Number of painful sites were selected that maximized sensitivity while maintaining a minimum specificity of 0.75 for predicting PPT gain-of-function.^54^

Unless otherwise stated, results are reported in the main text for primary analyses using PPTs (after log-transformation) at the proximal tibia distal to the participant's worst affected knee, taken to be an index for centrally augmented pain.^73^ Results for secondary analyses using PPT measured at other sites are reported within the supplementary tables (available online at http://links.lww.com/PAIN/A543).

### 2.4. Item selection

**Figure 1.**
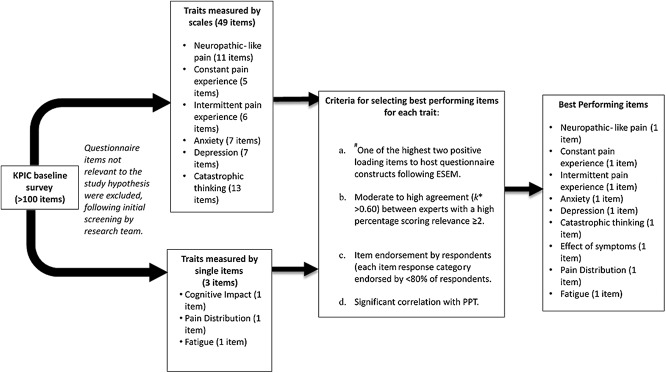
Flow chart showing the item selection process across traits. ESEM, exploratory structural equation modelling; PPT, pressure pain detection threshold. #Only relevant for items originating from established questionnaires measuring specific traits.

We used a sequential strategy to select items representing traits reflecting central pain mechanisms (Fig. 1):

(1) Items not relevant to the study hypothesis were excluded, after initial screening by the research team.
(2) Where items originated from established questionnaires (PCS, HADS, painDETECT, and ICOAP), the 2 items were selected with highest loading to each questionnaire's latent constructs. Item loading was determined by exploratory structural equation modelling (ESEM)^18^ across each questionnaire, using data from KPIC participants who reported current knee pain (n = 2152).
(3) Items were excluded if there was below moderate expert agreement (k* < 0.60) on their relevance to central mechanisms of knee pain.^12,27^ Invited experts comprised experienced clinical and research experts (n = 25) across various pain research disciplines (orthopaedics, rheumatology, sports and exercise medicine, psychology, neuroscience, physiotherapy, pharmacy, genetics, and musculoskeletal epidemiology) within the Arthritis Research UK (ARUK) Pain Centre. Experts indicated relevance for each item using a 4-point Likert scale (0 “not relevant” to 3 “highly relevant”).
(4) The percentage of respondents selecting each response category for an item was examined to ensure adequate targeting (a balanced frequency (%) of selection for each response category provided for an item across a study population). Items were excluded if any single response category was selected by ≥80% of participants.^8,47^
(5) Items were excluded if associations with PPT at the proximal tibia were not statistically significant. The PPT at the proximal tibia (an unaffected site, distal to the affected knee) was taken to be indicative of central pain mechanisms.^73^ Lack of a relationship between a self-report item and PPT was taken to indicate that the item might itself, not be indicative of central pain mechanisms.

### 2.5. Data analysis

Pressure pain detection threshold homogeneity was assessed using concordance correlation coefficient (CCC) to establish intrarater and interrater agreement for the 2 PPT assessors.^43^

Associations between PPT and questionnaire data in participants with knee pain (n = 322) are presented as Spearman correlation coefficients (*r*) or standardized regression coefficients (β) from linear regression models. Adjusted *P* values were obtained using Bonferroni correction. All analyses used complete case data because of low levels of missing data.

#### 2.5.1. Validation of selected items

For factor analysis of the selected items, participants with knee pain who had undergone PPT assessment (n = 322) were randomly allocated into 2 equal groups using Stata, version 14.2,^70^ to avoid spurious or chance effects.^28^ Exploratory structural equation modelling was used with 1 group and the resulting model was tested in the other group using confirmatory factor analysis (CFA). Pressure pain detection threshold variance explained by the identified factor(s) in fully adjusted models (adjusted for age, sex, and body mass index [BMI]) were compared with the variance explained by the host scales. To explore equivalence of the identified factor(s) and selected items with respect to age, sex, and BMI, Multiple-Indicator Multiple-Causal (MIMIC) models were used. MIMIC models are a type of CFA model where the latent factors and the items are simultaneously regressed on to demographics and other relevant covariates.^57^

We further sought to determine whether traits represented by the host scale explained the associations between PPT and items selected from that scale. Derived scale scores for each host scale were calculated by subtracting “the score for each selected item” from “the summary score for the respective host scale.” Each model testing the association between PPT and a selected item, or between PPT and any identified factor(s), was adjusted for derived scale scores.

Analyses were performed using Stata, version 14.2,^70^ except that ESEM and CFA used MPlus, version 7.4.^52^ Except where stated, all analyses were conducted within the participant group that reported knee pain and who had undergone PPT assessment (n = 322). Demographics are presented as mean (SD) or median (interquartile range). Between-group comparisons used Student *t* test and, where appropriate, 95% confidence intervals (CIs) are presented.

## 3. Results

### 3.1. Study population

The 322 participants with knee pain were on average 59 (SD 10) years of age, had an average BMI of 29 (SD 7), and most were female (61%). Participants without knee pain (n = 98, 60% female, age 60 ± 10 years) displayed geometric mean PPT at the proximal tibia of 383 (95% CI 169-780) kPA, similar to those with knee pain (358 [95% CI 134-871] kPa, *P* = 0.27).

Demographic and clinical characteristics for the knee pain group are presented in Table 1.

**Table 1:**
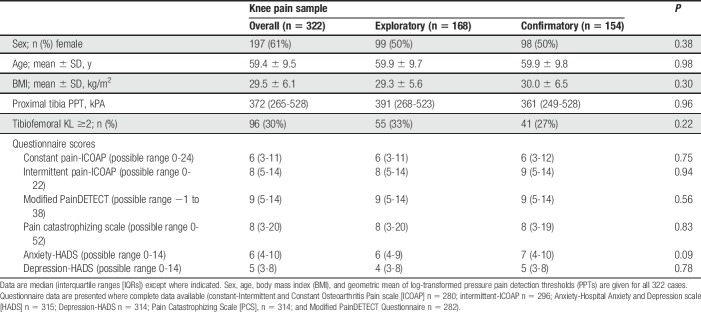
Baseline demographics and clinical characteristics of participants with knee pain.

### 3.2. Pressure pain detection thresholds

Pressure pain detection thresholds at the proximal tibia displayed moderate interrater reliability (CCC = 0.51) and intrarater reliability (CCC = 0.60) (Supplementary Table 1, available online at http://links.lww.com/PAIN/A543). Lower PPTs were associated with female sex (females; 314 [287-343] kPa, males; 428 [391-473] kPa, *P* < 0.0001) and higher BMI (*r* = −0.19, *P* = 0.002), but not with age (*r* = −0.01, *P* = 0.83). For those with knee pain, PPT was not associated with radiographic x-ray scores (*r* = −0.041, *P* = 0.491), but was associated with a painDETECT measure of knee pain severity (“How would you rate your most painful knee pain on a 0 to 10 scale at the present time, ie, right now”) (*r* = −0.18, *P* = 0.002). Pain severity showed a weak but significant relationship with radiographic scores (*r* = 0.15, *P* = 0.007).

#### 3.2.1. Pain distribution

The number of other sites reported as painful in addition to knee pain was negatively correlated with PPT distal to the index knee (23 other sites: *r* = −0.16, *P* = 0.008; 7 other sites: *r* = −0.16, *P* = 0.007). Cutoff points of ≥5/7 or ≥6/23 painful sites additional to knee, optimally predicted low PPT (specificity >0.75 and accuracy 73.4%). “Knee pain plus other pain below the waist” showed significant association with PPT (β = −0.14; *P* < 0.02), but other pain distribution categories did not (Table 2). ACR WSP classification did not significantly predict PPT, whether including (β = −0.03, *P* = 0.55) or excluding (β = −0.05; *P* = 0.37) knees as painful sites. The presence of “knee pain plus other pain below the waist” was selected for further analyses over “number of sites” criteria because of ease of application.

**Table 2:**
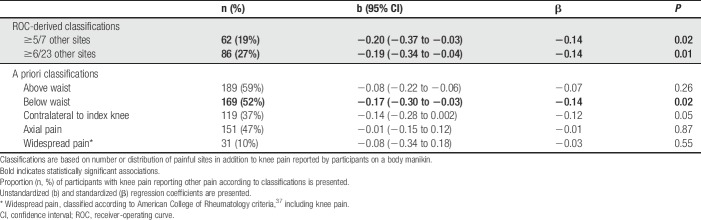
Pressure pain detection thresholds (PPTs) at the proximal tibia are predicted by ROC- and a priori-binary manikin classifications in individuals within the knee pain sample (n = 322).

### 3.3. Item selection

Twenty-five items potentially reflecting central mechanisms were selected for expert review. Exploratory structural equation modelling confirmed 11 latent factors from 4 questionnaires, representing anxiety or depression (HADS), magnification or rumination (PCS), pain intensity, evoked or spontaneous neuropathic-like pain (painDETECT), and psychological or somatic effects of pain (both in each of the ICOAP Constant and Intermittent ICOAP subscales) (Supplementary Tables 2–6, available online at http://links.lww.com/PAIN/A543). Two items were selected with highest loading to each of these factors. Additional items measured traits of fatigue, cognitive impact, and pain distribution (pain manikin). Sixteen (64%) experts responded to the consensus task and displayed moderate to excellent agreement (k > 0.6) for relevance of 19 of the 25 items to central pain mechanisms (Table 3).

**Table 3:**
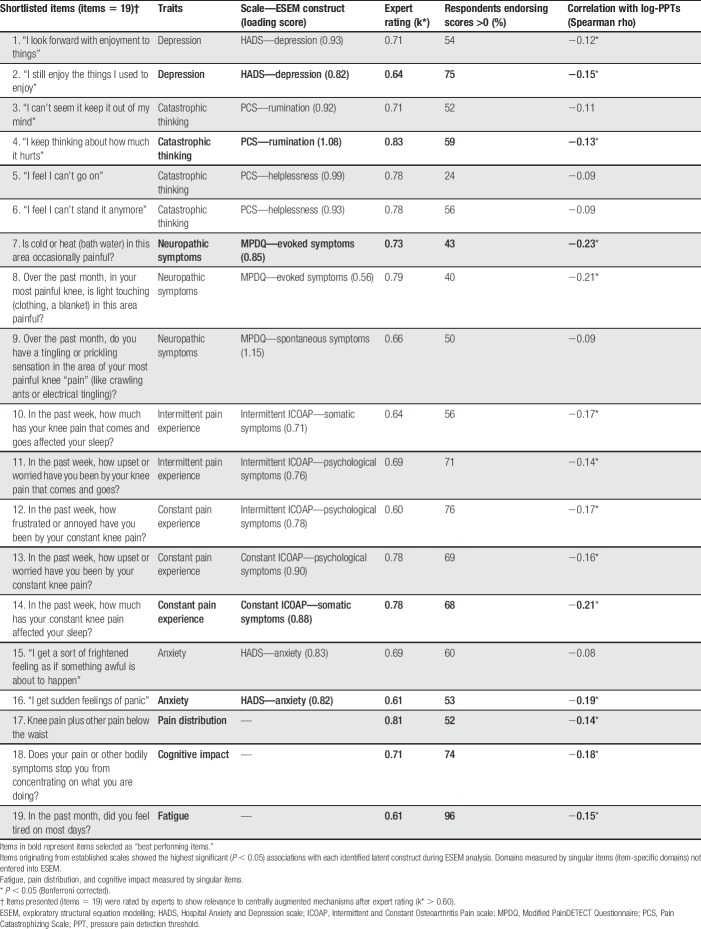
Item performance for each statistical criteria to select “best performing items” across traits.

Supplementary Table 7 gives item response distributions in people with knee pain (available online at http://links.lww.com/PAIN/A543). Each scale was positively associated with scores on other scales (*r* = 0.23-0.63, *P* < 0.05, Supplementary Table 8, available online at http://links.lww.com/PAIN/A543). The 19 items selected after expert review also all displayed significant positive associations with each other (*r* = 0.07-0.87, Supplementary Table 9, available online at http://links.lww.com/PAIN/A543). Items from the intermittent ICOAP subscale showed strong correlations (*r* > 0.8, *P* < 0.05) with corresponding constant ICOAP items.

#### 3.3.1. Association between pressure pain detection threshold and self-report scales or items

Each scale was negatively associated at a univariate level with PPT (β = −0.09 to −0.21, each *P* < 0.05 except intermittent-ICOAP, *P* = 0.13). A significant proportion of variation in PPT was explained by each scale alone (*R*^2^ values = 0.10-0.13, *P* < 0.05). Individual items displayed negative associations with PPT (Table 3). After excluding intermittent pain (to avoid item redundancy), a single item was selected to represent each of 8 remaining traits: fatigue, cognitive impact, pain distribution, anxiety, depression, catastrophic thinking, neuropathic-like, and constant pain (Table 3).

### 3.4. Validation of selected items

The 8 selected items displayed a Cronbach alpha (α) of 0.80, and predicted proximal tibia PPT in a multiple regression model (*R*^2^ = 0.18, *P* < 0.05) more than did any trait specific scale or item. Competing 2- and 3- factor models for these items were not identified in the exploratory group and a specified 2-factor CFA models did not significantly alter the 1-factor model, supporting the 1-factor model. The 1-factor model also showing the best fit to data from the Confirmatory group (root-mean-square error of approximation = 0.07; weighted root-mean-square residual = 0.5; X^2^(*df*) = 43(20)). Each item was significantly associated with the single latent construct, interpreted as representing central mechanisms of knee pain (Table 4).

**Table 4:**
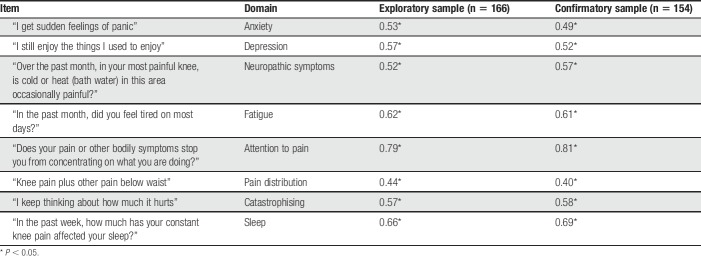
Standardized item loadings for the 8 selected items in a single factor model in exploratory and confirmatory subgroups.

The latent construct was associated with PPT (β = −0.27; SE = 0.07; *P* < 0.001), independent of each scale from which items were derived (Table 5). Associations between each selected item and PPT were reduced and lacked significance after adjusting for derived host scale scores (Supplementary Table 10, available online at http://links.lww.com/PAIN/A543), except for the neuropathic item on cold or heat on the area causing pain (β = −0.21, SE = 0.08, *P* < 0.05) and the anxiety item “I get sudden feelings of panic” (β = −0.19, SE = 0.09, *P* < 0.05), where the relationship remained significant after adjusting for derived host scale scores.

**Table 5:**
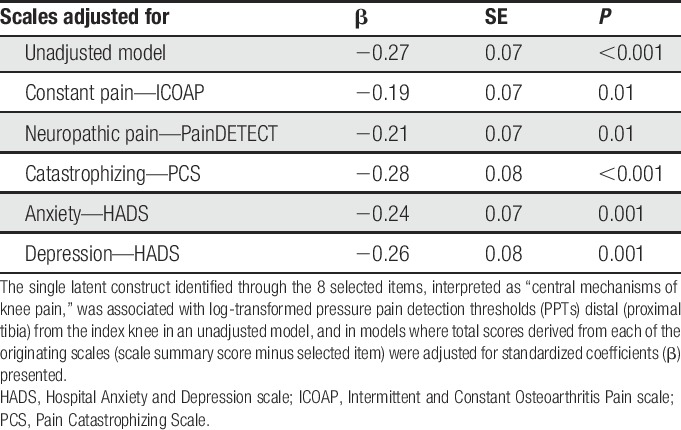
Prediction of proximal tibia PPT by identified factor independent of derived host scale scores (host scale score minus selected items score).

The latent construct explained a higher proportion of PPT variance at the proximal tibia (*R*^2^ = 0.17, SE = 0.05, *P* < 0.001), compared with that explained by any multi-item, trait-specific questionnaire (*R*^2^ values = 0.10-0.13, *P* < 0.05). The latent construct also explained a high proportion of PPT variance at the sternum (*R*^2^ = 0.20, SE = 0.05, *P* < 0.001), medial- (*R*^2^ = 0.34, SE = 0.05, *P* < 0.001), and lateral- (*R*^2^ = 0.24, SE = 0.05; *P* < 0.001) joint line. The latent construct was also associated with knee pain severity (β = 0.66; SE = 0.05, *P* < 0.001), but not radiographic scores (β = 0.10; SE = 0.07; *P* = 0.160). The relationship between the latent construct and PPT remained significant even when radiographic scores, or pain severity, were accounted for within the model (β = −0.267; SE = 0.07; *P* < 0.001, and β = −0.213; SE = 0.06; *P* < 0.001, respectively).

The final best-fitting MIMIC model was a good fit to the data (comparative fit index = 0.943, Tucker–Lewis index = 0.924; root-mean-square error of approximation = 0.050; weighted root-mean-square residual = 0.761; x^2^(*df*) = 53.696 (33)). An effect of BMI on the latent construct (β = 0.310, SE = 0.064, *P* < 0.001), but not sex (β = 0.073, SE = 0.070, *P* = 0.295) nor age (β = −0.064, SE = 0.069, *P* = 0.357), was observed. Item-specific effects for age (anxiety item: β = −0.114, SE = 0.055, *P* = 0.038) and BMI (depression item: β = 0.135, SE = 0.056, *P* = 0.015) were observed, but not for sex.

All secondary analyses using PPT at the index knee joint line or sternum produced similar results to those using proximal tibia PPT (Supplementary Tables 11 and 12, available online at http://links.lww.com/PAIN/A543).

## 4. Discussion

In the current study, we identified 8 key traits, represented by 8 self-report items which together load onto a single construct interpreted as reflecting central pain mechanisms in people with knee pain. The 8 key traits were anxiety, depression, catastrophizing, neuropathic-like pain, fatigue, sleep disturbance, pain distribution, and cognitive impact. Items representative of these traits displayed high face validity based on expert opinion and external validity by association with high pain sensitivity (low PPT) at a site distal to the index knee, indicative of central sensitization.^31^ These items might identify people whose knee pain could benefit from treatments directed towards central mechanisms.

Consistent with previous studies, we show that in individuals with knee pain, associations exist between reduced PPTs and increased scores on each of the 8 traits.^7,30,45^ Scores for each trait were significantly correlated with the other traits, consistent with a single latent construct, but a combination of the 8 traits explained more variation in PPTs compared with any originating questionnaire alone. We conclude that a combination of items from across these 8 traits might indicate the extent of central pain augmentation in people with knee pain. Consistent with previous reports where between 5% and 20% of PPT variance was explained by demographic, psychological, and/or genetic variables,^19,76^ the latent construct explains a significant proportion of PPT variance. This provides evidence of validity as a model of central sensitisation, but further research would be required to determine whether the identified construct explains a greater proportion of variation in other indices of central sensitisation, or variation in pain relief in response to interventions that target central sensitisation in people with knee pain.^66^

Augmented central pain processing is well recognised in people with chronic WSP but can be more difficult to identify when pain is focussed on a specific anatomical site such as the knee. Further research might define whether the traits identified in the current study of people with knee pain, might also reflect augmented central pain processing in people with pain at another site. Several items identified in this study represent the emotional component of pain, and shared mechanisms within the CNS might underpin associations with central pain augmentation.^48,71^ Cognitive difficulties or “brain fog” are frequent complaints of people with musculoskeletal pain,^50^ and experimental pain impairs performance in cognitive tasks.^20,75^ Neuropathic-like pain is also prevalent in people reporting knee OA pain and has been associated with reduced PPTs.^39,49^ Sleep disruption can lead to augmented central pain processing,^34^ and fatigue is strongly associated with musculoskeletal pain severity.^67^ Association between WSP and central mechanisms has been described previously.^10^ We extend these findings to show that higher numbers of painful sites, and pain below the waist other than knee pain, were each associated with reduced PPT. A minority of participants in our study satisfied ACR criteria for WSP and we might have lacked sufficient power to detect associations of WSP with PPT. However, our data indicate that central mechanisms might still contribute to pain in people with multisite pain who do not satisfy classification criteria for WSP.

Strength of association between each selected item and PPT was reduced after adjustment for originating questionnaire-derived score, suggesting at least partial mediation by the host construct. However, associations between PPT and items addressing neuropathic-like pain in response to cold or heat, or addressing feelings of panic remained statistically significant even after adjustment for the derived painDETECT and HADS-anxiety scores. These items might have specific associations with central mechanisms over and above representing neuropathic-like pain or anxiety, respectively.

The “central mechanisms” construct identified here explains slightly more PPT variance than that explained by any of the individual traits. Association between PPTs and the “central mechanisms” construct was found to be not explained by originating questionnaire-derived scores, disease severity, or pain severity. Together, these findings support use of a composite tool to identify the extent of central pain augmentation in people with knee pain rather than individual assessment of each trait on a case-by-case basis in clinical practice. Identification of these central pain mechanisms might well have prognostic relevance, and further work should assess whether central pain mechanisms might at least in part, explain the predictive values of other prognostic tools such as the Orebro Musculoskeletal Pain Screening Questionnaire,^44^ or StartBACK.^38^ Items reflecting psychological distress, similar to those included in the current study, are included within these scales. However, the Orebro and StartBACK questionnaires do not assess other key traits that we have identified in the current study, such as somatic traits of neuropathic-like symptoms and pain distribution.

Associations between the “central mechanisms” construct and increased BMI during MIMIC analysis support previous work in other chronic pain conditions, which demonstrate significant associations between BMI and other markers of central pain mechanisms.^26,58^ Addressing central pain mechanisms using nonpharmacological and/or pharmacological approaches is likely to improve pain treatment response, physical function, and other important outcomes for the individual.^32^ Further research should explore whether the core construct discovered here can predict pain outcome or response to treatment or help improve health care efficiency by directing targeted treatments. Randomized control trials might explore responsiveness of individuals with knee pain to novel or repurposed pharmacological and nonpharmacological therapies targeted to traits of psychological distress, neuropathic-like pain, and somatic disturbances identified in the current work.^21^ Longitudinal research might explore whether traits, or the central construct identified in the current study might predict better treatment response to such centrally targeted treatments. Conversely, traits identified in this study might indicate a central knee pain component which might not necessarily respond to a treatment that targets peripheral nociceptive drive.^48^ High catastrophizing predicted worse pain improvement after total knee arthroplasty in a previous study.^62^

This study is not without its limitations. Participant selection within KPIC for PPT assessments was weighted towards an early knee pain sample (pain for <3 years), and a high proportion had radiographic Kellgren and Lawrence scores <2. Previous studies have demonstrated a lack of association between PPTs and symptom duration in individuals with OA knee pain,^55^ but further research should determine whether our findings can be generalised to people with longer symptom duration or more severe OA structural change. The traits analysed were limited to those included within the KPIC baseline survey, and initial screening by the researchers may have allowed subjective bias during the initial stage of item selection. All experts involved within the current study originated from a single centre in the United Kingdom. Their breadth of expertise reflected multiple disciplines involved in the treatment and research of knee pain, but it is possible that additional traits might further contribute to the identification of pain mechanisms in people with knee pain. The current work is also limited because of the cross-sectional approach used, and longitudinal studies might help disentangle the nature of the relationship between pain severity, peripheral pathology, PPTs, and traits identified in the current study.

We used only 1 modality of quantitative sensory testing assessment—PPT—which was both used for item selection and other validation analysis. The PPT has consistently been associated with knee pain in previous studies and displays good measurement properties in people with knee pain.^53^ Our study design selected proximal tibia PPT, distal to the index knee, as a primary outcome index of central sensitisation. Index knee joint line PPT displayed higher reliability than proximal tibia PPT, but is likely to be dependent on peripheral and central sensitization.^55^ Pressure pain detection thresholds at remote sites displayed lower reliability than other sites and are less strongly associated with OA pain when compared with PPTs from sites distal to the affected joint.^55,73^ Further work is needed to confirm the specific central pathways that drive distal and remote pain sensitivity in knee OA.

Previous work has demonstrated associations between other modalities for accessing central pain mechanisms (eg, temporal summation or brain imaging), and self-report questionnaires about pain distribution, neuropathic-like symptoms, catastrophizing, sleep disturbance, fatigue, depression, and anxiety.^1,9,13,17,45^ These other modalities for assessing central mechanisms, especially those with higher reliability than PPTs, might produce more confident estimates of associations with the construct identified here.^41^

Further research should determine whether the central construct identified in the current study might also predict these other indices of central pain mechanisms. Central mechanisms and their self-report correlates present across a spectrum, rather than dichotomous presence or absence, and further research should define clinical thresholds that might predict or represent important response to treatment.

In conclusion, we show that 8 individual phenotypic traits, as well as a single overall construct (interpreted as “central pain mechanisms”) represented by 8 items, are correlates of a PPT index for centrally augmented pain in individuals with knee pain. These items might be combined to identify the extent of central pain augmentation in people with knee pain. Future research should determine whether a “central pain mechanisms” questionnaire can predict prognosis or treatment responses in people who present in a clinical setting with a local pain problem such as knee pain.

## Conflict of interest statement

W. Zhang: Consultation fees: AstraZeneca (Lesinurad) and Gruenthal (Lesinurad); Speaker fees: Husin (Chinese Society of Rheumatology Annual Congress 2016) and Bioberica (EULAR 2016 symposium) in the past 3 years. D.A. Walsh: Grants from Arthritis Research UK, during the conduct of the study; grants from Pfizer Ltd, other from Pfizer Ltd, personal fees from GlaxoSmithKline, outside the submitted work. The remaining authors have no conflicts of interest to declare.

This work was supported by Arthritis Research UK (Centre initiative grant number = 20777), and University of Nottingham as sponsor and host institution.

## Supplementary Material

SUPPLEMENTARY MATERIAL

## Appendix A. Supplemental digital content

Supplemental digital content associated with this article can be found online at http://links.lww.com/PAIN/A543.
