# Supplementary material for: Traits associated with central pain augmentation in the Knee Pain In the Community (KPIC) cohort
Source: Pain. 2018 Feb 9;159(6):1035–44. doi: 10.1097/j.pain.0000000000001183 (PMC5959005; doi:10.1097/j.pain.0000000000001183)
Supplement: SUPPLEMENTARY MATERIAL [file jop-159-1035-s001.docx]

**Supplementary Table 1. Inter- and intra- observer agreement for pressure pain detection threshold (PPT) assessments**

List of assessors: Laura Marshall (LM), Nadia Frowd (NF).

Abbreviations: CCC – concordance correlation coefficient, CI – confidence interval, PPT – pain pressure thresholds, n=number of participants assessed

|  | Inter-observer agreement | | | Intra-observer agreement | | |
| --- | --- | --- | --- | --- | --- | --- |
| Anatomical site | Observers | n | CCC (95% C.I.) | Observers | n | CCC (95% C.I.) |
| Proximal tibia | NF-LM | 8 | 0.51 (-0.01 to 1.02) | LM | 8 | 0.60 (0.28 to 0.92) |
| Sternum | NF-LM | 8 | 0.61 (0.17 to 1.05) | LM | 9 | 0.39 (-0.13 to 0.91) |
| Medial Joint Line | NF-LM | 8 | 0.75 (0.43 to 1.07) | LM | 8 | 0.90 (0.76 to 1.05) |
| Lateral Joint Line | NF-LM | 8 | 0.86 (0.68 to 1.03) | LM | 8 | 0.61 (0.10 to 1.13) |

**Supplementary Table 2: Standardized item loading for the Hospital Anxiety and Depression Scale (HADS) two factor model.**

| Items | Factor 1 (Depression) | Factor 2 (Anxiety) |
| --- | --- | --- |
| 1. I feel tense or wound up | 0.245*** | 0.603*** |
| 1. I still enjoy the things I used to enjoy | **0.840***** | 0.177** |
| 1. I get a sort of frightened feeling as if something awful is about to happen | 0.004 | **0.836***** |
| 1. I can laugh and see the funny side of things | 0.759*** | 0.054 |
| 1. Worrying thoughts go through my mind | 0.045 | 0.816*** |
| 1. I feel cheerful | 0.739*** | 0.074 |
| 1. I can sit at ease and feel relaxed | 0.629*** | 0.205*** |
| 1. I feel as if I am slowed down | 0.129 | 0.226*** |
| 1. I get a sort of frightened feeling like ‘butterflies’ in the stomach | 0.092 | 0.484*** |
| 1. I have lost interest in my appearance | 0.592*** | 0.008 |
| 1. I feel restless as if I have to be on the move | 0.112* | 0.419*** |
| 1. I look forward with enjoyments to things | **0.943***** | -0.109 |
| 1. I get sudden feelings of panic | 0.031 | **0.868***** |
| 1. I can enjoy a good book or radio or television programme | 0.591*** | 0.028 |

Fit statistics for two factor model: CFI = 0.985; TLI=0.979; RMSEA = 0.073; WRMR = 3.127; X^2^(df) = 220 (64).

Items in bold represents the two highest loading items within each identified factor.

* p<0.05 **p<0.01 *** p<0.001

**Supplementary Table 3: Standardized item loading for the Pain Catastrophizing Scale (PCS) two factor model.**

| Items | Factor 1  (Helplessness) | Factor 2  (Rumination) |
| --- | --- | --- |
| 1. I worry all the time about whether the pain will end | 0.470*** | 0.395*** |
| 1. I feel I can‘t go on | **1.000***** | 0.131* |
| 1. It’s terrible and I think it’s never going to get any better | 0.858*** | 0.074 |
| 1. It’s awful and I feel that it overwhelms me | 0.871*** | 0.101** |
| 1. I feel I can’t stand it anymore | **0.932***** | 0.001 |
| 1. I become afraid that the pain will get worse | 0.495*** | 0.384*** |
| 1. I keep thinking of other painful events | 0.460*** | 0.361*** |
| 1. I anxiously want the pain to go away | 0.069 | 0.814*** |
| 1. I can’t seem it keep it out of my mind | 0.000 | **0.921***** |
| 1. I keep thinking about how much it hurts | 0.169*** | **1.097***** |
| 1. I keep thinking about how badly I want the pain to stop | 0.041 | 0.901*** |
| 1. There’s nothing I can do to reduce the intensity of the pain | 0.310*** | 0.542*** |
| 1. I wonder whether something serious may happen | 0.240** | 0.519*** |

Fit statistics for two factor model: CFI = 0.998; TLI=0.997; RMSEA = 0.043; WRMR = 1.183; X^2^(df) = 253 (53)

Items in bold represents the two highest loading items within each identified factor.

* p<0.05 **p<0.01 *** p<0.001

**Supplementary Table 4: Standardized item loading for the PainDETECT (PDQ) three factor model.**

| Items | Factor 1  (Pain Intensity) | Factor 2  (Spontaneous Neuropathic- Like pain) | Factor 3  (Evoked Neuropathic- Like pain) |
| --- | --- | --- | --- |
| 1. Over the past month, does your pain run up and down your leg? | 0.218*** | 0.424*** | 0.033 |
| 1. How would you rate your most painful knee pain on a 0-10 scale at the present time, that is right now, where 0 is ‘no pain’ and 10 is ‘pain as bad as could be’? | 0.664*** | 0.063 | 0.109* |
| 1. In the past month. How intense was your worst knee pain rated on a 0-10 scale, where 0 is ‘no pain’ and 10 is ‘pain as bad as could be’? | **0.926***** | 0.006 | 0.005 |
| 1. In the past month, on average, how intense was the pain in your most painful knee rated on a 0-10 scale, where 0 is ‘no pain’ and 10 is ‘pain as bad as could be’? | **0.959***** | 0.005 | 0.023 |
| 1. The next question is on the pattern of your pain in your most painful knee. Which of the 4 different options below is the one that best describes the pattern of your worst knee pain over the past month? | -0.283*** | 0.043 | 0.273 |
| 1. Do you suffer from a burning sensation (e.g., stinging nettles) in or around your most painful knee? | 0.100* | **0.710***** | 0.007*** |
| 1. Do you have a tingling or prickling sensation in the area of your most painful knee ‘pain’ (like crawling ants or electrical tingling)? | 0.001 | **1.153***** | 0.226* |
| 1. Is light touching (clothing, a blanket) in this area painful? | 0.149** | 0.191*** | **0.559***** |
| 1. Do you have sudden pain attacks in the area of your pain, like electric shocks? | 0.237*** | 0.240*** | 0.177** |
| 1. Is cold or heat (bath water) in this area occasionally painful? | 0.001 | 0.002 | **0.861***** |
| 1. Do you suffer from a sensation of numbness in the areas that you marked? | 0.102* | 0.400*** | 0.361*** |
| 1. Does slight pressure in this area, e.g., with a finger, trigger pain? | 0.152 | 0.001 | 0.520*** |

Fit statistics for two factor model: CFI = 0.987; TLI=0.974; RMSEA = 0.038; WRMR = 0.54; X^2^(df) = 65.41 (33)

Items in bold represents the two highest loading items within each identified factor.

* p<0.05 **p<0.01 *** p<0.001

**Supplementary Table 5: Standardized item loading for the Constant pain ICOAP two factor model.**

| Items | Factor 1  (Somatic effects of constant pain) | Factor 2  (Psychological effects of constant pain) |
| --- | --- | --- |
| 1. In the past week, how intense has your *constant knee pain* been? | **0.797*** | 0.165 |
| 1. In the past week, how much has your *constant knee pain* affected your sleep? | **0.888*** | 0.005 |
| 1. In the past week, how much has your *constant knee pain* affected your overall quality of life? | 0.417* | 0.552* |
| 1. In the past week, how frustrated or annoyed have you been by your *constant knee pain*? | 0.184 | **0.780*** |
| 1. In the past week, how upset or worried have you been by your *constant knee pain*? | 0.006 | **0.940*** |

Fit statistics for two factor model: CFI = 1.00; TLI=1.00; RMSEA = 0.014; WRMR = 0.106; X^2^(df) = 1.15 (1)

Items in bold represents the two highest loading items within each identified factor.

*p < 0.001

**Supplementary Table 6: Standardized item loading for the Intermittent pain ICOAP two factor model.**

| Items | Factor 1  (Somatic effects of constant pain) | Factor 2  (Psychological effects of constant pain) |
| --- | --- | --- |
| 1. In the past week, how intense has your knee pain that comes and goes been? | **0.967**** | 0.003 |
| 1. In the past week, how much has your knee pain that comes and goes affected your sleep? | **0.709**** | 0.246* |
| 1. In the past week, how much has your knee pain that comes and goes affected your overall quality of life? | 0.485** | 0.380** |
| 1. In the past week, how frustrated or annoyed have you been by your knee pain that comes and goes? | 0.379** | 0.601** |
| 1. In the past week, how upset or worried have you been by your knee pain that comes and goes? | 0.215* | **0.758**** |
| 1. In the past week, how frequently has this knee pain that comes and goes occurred? | 0.006 | **0.964**** |

Fit statistics for two factor model: CFI = 1.00; TLI=0.99; RMSEA = 0.06; WRMR = 0.353; X^2^(df) = 15.8 (4)

Items in bold represents the two highest loading items within each identified factor.

* p<0.05** p < 0.001

**Supplementary Table 7. Responses from participants with knee pain to items selected as relevant to central pain mechanisms**

| Domains | Items | knee pain sample | | | p |
| --- | --- | --- | --- | --- | --- |
|  |  | **Overall**  **(n = 322)** | **Exploratory**  **(n = 168)** | **Confirmatory**  **(n = 154)** |  |
| Anxiety | I get sudden feelings of panic (possible range 0 to 3) | 1 (0 to 1) | 1 (0 to 1) | 1 (0 to 1) | 0.204 |
|  | I get a sort of frightened feeling as if something awful is about to happen ( possible range 0 to 3) | 1 (0 to 2) | 1 (0 to 2) | 1 (0 to 2) | 0.199 |
| Depression | I still enjoy the things I used to enjoy (possible range 0 to 3) | 1 (0 to 1) | 1 (0 to 1) | 1 (1 to 1) | 0.887 |
|  | I look forward with enjoyments to things (possible range 0 to 3) | 1 (0 to 1) | 1 (0 to 1) | 1 (0 to 1) | 0.746 |
| Neuropathic- like pain | Do you have a tingling or prickling sensation in the area of your most painful knee ‘pain’ (like crawling ants or electrical tingling)? (possible range 0 to 5) | 1 (0 to 2) | 0 (0 to 2) | 1 (0 to 2) | 0.991 |
|  | Is light touching (clothing, a blanket) in this area painful?  (possible range 0 to 5) | 0 (0 to 1) | 0 (0 to 1) | 0 (0 to 2) | 0.832 |
|  | Is cold or heat (bath water) in this area occasionally painful?  (possible range 0 to 5) | 0 (0 to 1) | 0 (0 to 1) | 0 (0 to 1) | 0.984 |
| Fatigue | In the past month, did you feel tired on most days?  (possible range 0 to 5) | 2 (2 to 3) | 2 (2 to 3) | 2 (2 to 3) | 0.352 |
| Cognitive Impact | Does your pain or other bodily symptoms stop you from concentrating on what you are doing? (possible range 0 to 4) | 1 (0 to 2) | 2 (1 to 2) | 1 (0 to 2) | 0.481 |
| Pain Distribution | Other pain below waist (possible range 0 to 1) | 1 (0 to 1) | 1 (0 to 1) | 1 (0 to 1) | 0.793 |
| Pain Catastrophizing | I feel I can’t stand it anymore (possible range 0 to 4) | 0 (0 to 1) | 0 (0 to 1) | 0 (0 to 1) | 0.359 |
|  | I feel I can‘t go on (possible range 0 to 4) | 0 (0 to 0) | 0 (0 to 1) | 0 (0 to 0) | 0.415 |
|  | I can’t seem it keep it out of my mind (possible range 0 to 4) | 1 (0 to 2) | 1 (0 to 2) | 1 (0 to 1) | 0.423 |
|  | I keep thinking about how much it hurts (possible range 0 to 4) | 1 (0 to 2) | 1 (0 to 2) | 1 (0 to 2) | 0.788 |
| Constant pain experience | In the past week, how much has your *constant knee pain* affected your sleep? (possible range 0 to 4) | 1 (0 to 2) | 1 (0 to 2) | 1 (0 to 2) | 0.504 |
|  | In the past week, how frustrated or annoyed have you been by your *constant knee pain*? (possible range 0 to 4) | 1 (1 to 3) | 1 (1 to 3) | 2 (0 to 3) | 0.792 |
|  | In the past week, how upset or worried have you been by your *constant knee pain*? (possible range 0 to 4) | 1 (0 to 2) | 1 (0 to 2) | 1 (0 to 2) | 0.651 |
| Intermittent pain experience | In the past week, how much has your knee pain that comes and goes affected your sleep? (possible range 0 to 4) | 1 (0 to 2) | 1 (0 to 2) | 1 (0 to 2) | 0.915 |
|  | In the past week, how upset or worried have you been by your knee pain that comes and goes? (possible range 0 to 4) | 1 (0 to 2) | 1 (0 to 2) | 1 (0 to 2) | 0.229 |

Data are median (interquartile ranges, IQR).

|  | Neuropathic- like pain – painDETECT | Constant Pain - ICOAP | Intermittent pain - ICOAP | Depression -HADS | Anxiety -HADS |
| --- | --- | --- | --- | --- | --- |
| Constant Pain – ICOAP | 0.63* | - | - | - | - |
| Intermittent pain – ICOAP | 0.62* | 0.62* | - | - | - |
| Depression – HADS | 0.39* | 0.43* | 0.32* | - | - |
| Anxiety- HADS | 0.33* | 0.30* | 0.23* | 0.57* | - |
| Pain Catastrophizing - PCS | 0.50* | 0.57* | 0.47* | 0.57* | 0.58* |

**Supplementary Table 8. Associations between self-report measures.**

ICOAP (Intermittent and Constant Osteoarthritis Pain), HADS (Hospital Anxiety and Depression scale), PCS (Pain Catastrophizing Scale). Data from participants with knee pain (n=322). Data are Spearman correlation coefficients using untransformed total scale scores. *P<0.05

**Supplementary Table 9. Inter-item correlation matrix for 19 items putatively reflecting central mechanisms in people with knee pain.**

| Domains |  | Anxiety | | Depression | | Neuropathic- like pain | | | Fatigue | Cognitive Impact | Pain Distribution | Pain Catastrophizing | | | | Constant pain experience | | | Intermittent pain experience |
| --- | --- | --- | --- | --- | --- | --- | --- | --- | --- | --- | --- | --- | --- | --- | --- | --- | --- | --- | --- |
|  | Items | Fright | Panic | Still enjoy | Look forward | Tingling | Light touch | Cold  heat | Tired | Concentrate on pain | Other pain below waist | Can’t stand it | Can’t go on | Out of mind | Keep thinking | Sleep | Frustrate | Upset | Sleep |
| Anxiety | Panic | 0.66* | 1.00 | - | - | - | - | - | - | - | - | - | - | - | - | - | - | - | - |
| Depression | Still enjoy | 0.19* | 0.20* | 1.00 | - | - | - | - | - | - | - | - | - | - | - | - | - | - | - |
|  | Look forward | 0.32* | 0.28* | 0.56* | 1.00 | - | - | - | - | - | - | - | - | - | - | - | - | - | - |
| Neuropathic- like pain | Tingling | 0.24* | 0.20* | 0.27* | 0.20* | 1.00 | - | - | - | - | - | - | - | - | - | - | - | - | - |
|  | Light touch | 0.22* | 0.29* | 0.26* | 0.23* | 0.51* | 1.00 | - | - | - | - | - | - | - | - | - | - | - | - |
|  | Cold/heat | 0.21* | 0.20* | 0.25* | 0.26* | 0.47* | 0.65* | 1.00 | - | - | - | - | - | - | - | - | - | - | - |
| Fatigue | Tired | 0.41* | 0.39* | 0.33* | 0.37* | 0.23* | 0.31* | 0.33* | 1.00 | - | - | - | - | - | - | - | - | - | - |
| Cognitive Impact | Concentrate on pain | 0.35* | 0.33* | 0.41* | 0.38* | 0.38* | 0.41* | 0.37* | 0.44* | 1.00 | - | - | - | - | - | - | - | - | - |
| Pain Distribution | Other pain below waist | 0.11 | 0.07 | 0.21* | 0.24* | 0.07 | 0.19* | 0.13* | 0.18* | 0.29* | 1.00 | - | - | - | - | - | - | - | - |
| Pain Catastrophizing | Can’t stand it | 0.41* | 0.34* | 0.35* | 0.43* | 0.42* | 0.29* | 0.33* | 0.36* | 0.49* | 0.11 | 1.00 |  | - | - | - | - | - | - |
|  | Can’t go on | 0.40* | 0.32* | 0.37* | 0.44* | 0.36* | 0.31* | 0.33* | 0.32* | 0.42* | 0.11 | 0.67* | 1.00 | - | - | - | - | - | - |
|  | Out of mind | 0.46* | 0.40* | 0.28* | 0.44* | 0.39* | 0.33* | 0.35* | 0.38* | 0.44* | 0.17* | 0.69* | 0.53* | 1.00 | - | - | - | - | - |
|  | Keep thinking | 0.44* | 0.39* | 0.22* | 0.41* | 0.35* | 0.38* | 0.34* | 0.33* | 0.42* | 0.19* | 0.71* | 0.51* | 0.78* | 1.00 | - | - | - | - |
| Constant pain experience | Sleep | 0.17* | 0.19* | 0.27* | 0.29* | 0.45* | 0.53* | 0.51* | 0.35* | 0.48* | 0.26* | 0.44* | 0.32* | 0.41* | 0.43* | 1.00 | - | - | - |
|  | Frustrate | 0.22* | 0.18* | 0.36* | 0.32* | 0.49* | 0.42* | 0.45* | 0.35* | 0.47* | 0.24* | 0.52* | 0.36* | 0.50* | 0.48* | 0.67* | 1.00 | - | - |
|  | Upset | 0.26* | 0.26* | 0.31* | 0.32* | 0.49* | 0.41* | 0.41* | 0.32* | 0.45* | 0.20* | 0.47* | 0.37* | 0.51* | 0.49* | 0.60* | 0.87* | 1.00 | - |
| Intermittent pain experience | Sleep | 0.24* | 0.26* | 0.26* | 0.30* | 0.48* | 0.53* | 0.48* | 0.33* | 0.46* | 0.26* | 0.46* | 0.33* | 0.42* | 0.44* | 0.85* | 0.63* | 0.59* | 1.00 |
|  | Upset | 0.26* | 0.26* | 0.29* | 0.33* | 0.49* | 0.37* | 0.42* | 0.31* | 0.45* | 0.21* | 0.48* | 0.39* | 0.53* | 0.51* | 0.50* | 0.78* | 0.87* | 0.54* |

Full item texts were the same as given in Supplementary Table 7. Data are Spearman correlation coefficients from participants with knee pain (n=322). *p<0.05.

**Supplementary Table 10. Associations between selected items and proximal tibia pressure pain detection threshold (PPT) are dependent on constructs measured by their host questionnaires.**

|  | Unadjusted model | | | Adjusted model |  |  |
| --- | --- | --- | --- | --- | --- | --- |
| Domains | b (95% CI) | β | p | b (95% CI) | β | p |
| Constant Pain Experience  “In the past week, how much has your constant knee pain affected your sleep?” | **-0.11 (-0.17 to -0.04)** | **-0.21** | **0.001** | -0.08 (-0.17 to 0.02) | -0.15 | 0.119 |
| Neuropathic- like pain  “Over the past month, in your most painful knee, is cold or heat (bath water) in this area occasionally painful?” | **-0.10 (-0.17 to -0.05)** | **-0.23** | **<0.001** | **-0.10 (-0.17 to -0.03)** | **-0.21** | **0.008** |
| Catastrophizing  "I keep thinking about how much it hurts" | **-0.06 (-0.12 to -0.01)** | **-0.13** | **0.03** | 0.004 (-0.12 to 0.12) | 0.007 | 0.953 |
| Anxiety  "I get sudden feelings of panic" | **-0.13 (-0.21 to -0.05)** | **-0.19** | **0.001** | **-0.12 (-0.24 to -0.01)** | **-0.19** | **0.032** |
| Depression  "I still enjoy the things I used to enjoy" | **-0.10 (-0.18 to -0.02)** | **-0.15** | **0.01** | -0.06 (-0.16 to 0.04) | -0.09 | 0.252 |

In order to explore whether observed univariate associations between each selected item and proximal tibia log-PPTs might be explained by the construct measured by the host scale from the host scale from which it originated, we adjusted each univariate association for the derived host scale score (scale summary score minus selected item). Data are from participants with knee pain sample (n=322). Bold indicates significant associations after adjustment. Unstandardized (b) and standardized coefficients (β) are presented.

Supplementary Table 11: PPTs at sites other than the proximal tibia are predicted by ROC- derived and *a priori*- binary manikin classifications in individuals within the knee pain sample (n=322)

|  | Sternum | | | Med JL | | | Lat JL | | |
| --- | --- | --- | --- | --- | --- | --- | --- | --- | --- |
|  | b (95% CI) | β | p | b (95% CI) | β | p | b (95% CI) | β | p |
| *Roc-Derived Classifications* |  |  |  |  |  |  |  |  |  |
| ≥5/7 other sites | **-0.20 (-0.37 to -0.03)** | **-0.18** | **0.002** | **-0.24 (-0.39 to -0.09)** | **-0.15** | **0.011** | **-0.29 (-0.47 to -0.12)** | **-0.19** | **0.001** |
| ≥6/23 other sites | **-0.19 (-0.34 to -0.04)** | **-0.14** | **0.019** | **-0.16 (-0.30 to -0.03)** | **-0.14** | **0.018** | **-0.21 (-0.36 to -0.05)** | **-0.15** | **0.010** |
| *A priori Classifications* |  |  |  |  |  |  |  |  |  |
| Above waist | -0.08 (-0.22 to -0.06) | -0.05 | 0.430 | -0.05 (-0.17 to 0.07) | -0.08 | 0.205 | -0.08 (-0.22 to 0.06) | -0.07 | 0.266 |
| Below waist | **-0.17 (-0.30 to -0.03)** | **-0.16** | **0.007** | **-0.27 (-0.42 to -0.12)** | **-0.21** | **0.001** | **-0.22 (-0.36 to -0.08)** | **-0.18** | **0.002** |
| Contralateral to index knee | -0.14 (-0.28 to 0.002) | -0.08 | 0.165 | -0.18 (-0.34 to 0.03) | **-0.14** | **0.021** | -0.12 (-0.27 to 0.02) | -0.09 | 0.100 |
| Axial pain | -0.01 (-0.15 to 0.12) | -0.05 | 0.441 | -0.08 (-0.23 to 0.07) | -0.06 | 0.318 | -0.07 (-0.21 to 0.07) | -0.06 | 0.309 |
| ACR’s Widespread pain^a^ | -0.10 (-0.34 to 0.14) | -0.05 | 0.407 | -0.09 (-0.39 to 0.20) | -0.04 | 0.533 | 0.01 (-0.22 to 0.25) | 0.007 | 0.910 |

Classifications are based on number or distribution of painful sites in addition to knee pain reported by participants on a body manikin. ^a^Widespread pain; classified according to American College of Rheumatology criteria^37^, including knee pain. Bold indicates statistically significant associations. ROC; receiver-operating curve. Log-transformed pressure pain detection thresholds (PPT) at (medial or lateral tibiofemoral joint line (JL), or remote (sternum) from the index knee reported here. Data utilized from knee pain sample (n=322). Unstandardized (b) and standardized (β) regression coefficients are presented.

Supplementary Table 12. Associations between latent construct `Central mechanisms’ and PPTs for sites other than proximal tibia within the knee pain sample (n=322)

| Scales adjusted for | Sternum | | | Medial JL | | | Lateral JL | | |
| --- | --- | --- | --- | --- | --- | --- | --- | --- | --- |
|  | β | S.E | P | β | S.E | P | β | S.E | P |
| Unadjusted Model | **-0.25** | **0.06** | **<0.001** | **-0.41** | **0.06** | **<0.001** | **-0.39** | **0.06** | **<0.001** |
| Constant Pain experience - ICOAP | **-0.22** | **0.07** | **0.001** | **-0.32** | **0.06** | **<0.001** | **-0.29** | **0.07** | **<0.001** |
| Neuropathic- like pain - PainDETECT | **-0.22** | **0.06** | **0.001** | **-0.31** | **0.07** | **<0.001** | **-0.30** | **0.07** | **<0.001** |
| Catastrophizing - PCS | **-0.21** | **0.07** | **0.003** | **-0.38** | **0.07** | **<0.001** | **-0.34** | **0.07** | **<0.001** |
| Anxiety - HADS | **-0.20** | **0.07** | **0.003** | **-0.37** | **0.06** | **<0.001** | **-0.35** | **0.06** | **<0.001** |
| Depression - HADS | **-0.19** | **0.07** | **0.008** | **-0.42** | **0.07** | **<0.001** | **-0.37** | **0.07** | **<0.001** |

The single latent construct identified through the 8 selected items, interpreted as `central mechanisms of knee pain’, was associated with log-transformed pressure pain detection thresholds (PPT) at (medial or lateral tibiofemoral joint line (JL), or remote (sternum) from the index knee in an unadjusted model, and in models where total scores derived from each of the originating scales (scale summary score minus selected item) were adjusted for.

Standardized regression coefficients (β) presented.
